# Effects of cordycepin on HepG2 and EA.hy926 cells: Potential antiproliferative, antimetastatic and anti-angiogenic effects on hepatocellular carcinoma

**DOI:** 10.3892/ol.2014.1965

**Published:** 2014-03-11

**Authors:** HAISHENG LU, XITING LI, JIANYING ZHANG, HUI SHI, XIAOFENG ZHU, XIAOSHUN HE

**Affiliations:** 1 Organ Transplantation Center, First Affiliated Hospital, Sun Yat-Sen University, Guangzhou, Guangdong 510080, P.R. China; 2 Department of Periodontology, Guanghua School of Stomatology, Sun Yat-Sen University, Guangzhou, Guangdong 510055, P.R. China; 3 Department of Radiology, The Affiliated Hexian Memorial Hospital, Southern Medical University, Guangzhou, Guangdong 511400, P.R. China

**Keywords:** cordycepin, angiogenesis, invasion, hepatocellular carcinoma, apoptosis, vascular endothelial cells

## Abstract

Hepatocellular carcinoma (HCC) is a hypervascular tumor and accumulating evidence suggests that angiogenesis plays an important role in HCC development. Cordycepin, also known as 3′-deoxyadenosine, is a derivative of adenosine, and numerous cellular enzymes cannot differentiate the two. The aim of the present study was to determine whether cordycepin regulates proliferation, migration and angiogenesis in a human umbilical vein endothelial cell line (EA.hy926) and in a hepatocellular carcinoma cell line (HepG2). MTT was used to assess cell proliferation. Apoptosis was analyzed by flow cytometry (propidium iodide staining). Transwell and wound healing assays were used to analyze the migration and invasion of HepG2 and EA.hy926 cells. Angiogenesis in EA.hy926 cells was assessed using a tube formation assay. Cordycepin strongly suppressed HepG2 and EA.hy926 cell proliferation in a dose- and time-dependent manner. Cordycepin induced EA.hy926 cell apoptosis in a dose-dependent manner (2,000 μg/ml: 50.20±1.55% vs. 0 μg/ml: 2.62±0.19%; P<0.01). Cordycepin inhibited EA.hy926 cell migration (percentage of wound healing area, 2,000 μg/ml: 3.45±0.29% vs. 0 μg/ml: 85.48±0.84%; P<0.05), as well as tube formation (total length of tubular structure, 1,000 μg/ml: 107±39 μm vs. 0 μg/ml: 936±56 μm; P<0.05). Cordycepin also efficiently inhibited HepG2 cell invasion and migration. High-performance liquid chromatography analysis of the cytosol from EA.hy926 cells showed that cordycepin was stable for 3 h. In conclusion, cordycepin not only inhibited human HepG2 cell proliferation and invasion, but also induced apoptosis and inhibited migration and angiogenesis in vascular endothelial cells, suggesting that cordycepin may be used as a novel anti-angiogenic therapy in HCC.

## Introduction

Hepatocellular carcinoma (HCC) is responsible for over 600,000 mortalities each year; it is the sixth most common type of cancer in the world and the third greatest cause of cancer mortality (1–3). HCC prognosis is generally poor and the 5-year survival rate is <7% (4). Surgical resection and liver transplantation are still recognized as effective curative approaches for HCC; however, they are possible in only a small number of patients. Eventually, the majority of patients exhibit intrahepatic recurrences that quickly progress to an advanced disease, with blood vessel invasion and multiple extrahepatic metastases (5). Angiogenesis is a complex process based on the activation, proliferation and migration of endothelial cells. During angiogenesis, endothelial cells are activated by angiogenic factors. The cells then secrete proteases to dissolve their basement membrane, allowing their migration toward the angiogenic signal, where they can proliferate and form new blood vessels (6). Uncontrolled cell proliferation and angiogenesis play critical roles in HCC growth, pathological classification, metastatic spread and prognosis (7).

Chemotherapy is often the only treatment for advanced and inoperable HCC. However, its outcomes are often discouraging due to poor tolerance and low efficacy (8). In the recent decade, natural products have been a rich source of compounds with numerous applications in cancer therapy, without the associated side effects. For these reasons, a number of researchers are trying to screen antitumor compounds from various natural substances. Cordycepin (3′-deoxyadenosine), is the major bioactive component of *Cordyceps militaris* (9) and is a natural structural analog of adenosine (10). Its pharmacokinetic profile indicates that the cordycepin-induced metabolite is suppressed by an adenosine deaminase inhibitor *in vivo*, and that it has a short half-life and high rates of clearance (11). This molecule was shown more than 40 years ago to have antitumor activities in rodent and human *in vivo* and *in vitro* systems (12–14). However, in previous studies, different sources and various concentrations of purified cordycepin affect the consistency of these conclusions. *In vitro*, cordycepin was tested in various cancer cells, including breast, prostate, colon, leukemia and lung carcinoma cells (15–20), as well as in hepatic cancer cells (21–24).

A number of previous studies have assessed the effects of cordycepin on HCC cells; however, the effects of cordycepin on vascular endothelial cell migration and angiogenesis require investigation. Furthermore, no information is available regarding the intracellular levels of cordycepin following treatment in endothelial cells. Therefore, the primary aim of the present study was to assess the antimigration and anti-angiogenic effects of cordycepin on vascular endothelial cells, and the stability of intracellular cordycepin levels following administration.

## Materials and methods

### Reagents

Professor Li from the South China Normal University (Guangzhou, China) developed a novel column chromatography extraction method for the extraction of cordycepin from solid rice-based fermentation medium. Using this method, cordycepin is obtained at a 98% purity, with an overall recovery rate of 90% (25). Cordycepin was provided by his laboratory and was freshly prepared as stock solution in double-distilled water. It was diluted in culture medium at concentrations of 125, 250, 500, 1,000 and 2,000 μg/ml prior to experiments. Dulbecco’s modified Eagle’s medium (DMEM) and 4′, 6-Diamidino-2-phenylindole (DAPI) were purchased from Invitrogen Corporation (Carlsbad, CA, USA). Fetal bovine serum (FBS) was purchased from Gibco Industries Inc. (Big Cabin, OK, USA) and the cell apoptosis propidium iodide (PI) detection kit was purchased from Nanjing KeyGen Biotechnology Co., Ltd. (Nanjing, China). 3-(4,5-Dimethylthiazol-2-yl)-2,5-diphenyltetrazoliumbromide (MTT) and dimethyl sulfoxide (DMSO) were purchased from Sigma-Aldrich (St. Louis, MO, USA). MTT was dissolved in phosphate-buffered saline (PBS; Gibco, Carlsbad, CA, USA) and stored in the dark. Transwell chamber and Matrigel were purchased from BD Biosciences (San Jose, CA, USA). All reagents were of analytical grade, unless otherwise specified.

### Cell culture

Human HCC cells (HepG2) and human endothelial-like immortalized cells (EA.hy926) were obtained from the Cell Bank of Type Culture Collection of Chinese Academy of Sciences (Shanghai, China). EA.hy926 and HepG2 cells were cultured in DMEM supplemented with 10% (v/v) heat-inactivated fetal calf serum, penicillin (100 U/ml) and streptomycin (100 U/ml) (both Sigma-Aldrich). Cultures were maintained at 37°C in a humidified atmosphere containing 5% CO_2_ and 95% air. The medium was changed every two days.

### Cell viability assay

Cell survival changes in response to cordycepin were evaluated by MTT assay (8,26). Briefly, 2×10^4^ cells in 100 μl DMEM supplemented with 2% (v/v) heat-inactivated FBS, penicillin (100 U/ml) and streptomycin (100 U/ml) were seeded into 96-well plates. Medium without cells was used as a blank control. Confluent cells were treated with various concentrations of cordycepin (125, 250, 500, 1,000 and 2,000 μg/ml) for 1, 2, 3, 4 and 5 days. The same volume of double-distilled water was used as the negative control (0 μg/ml). At the designed time points, 100 ml MTT solution in PBS was added to obtain a final concentration of 0.5 g/ml, and the incubation was continued at 37°C for 4 h. Finally, the medium was removed and replaced with 200 μl DMSO. The mixture was quantified by determining its absorbance at 540 nm using a SpectraFluor Plus Reader (Tecan AG, Hombrechtikon, Switzerland). The relative growth rate was calculated as optical density (OD)_test group_/OD_negative control_.

### Flow cytometric detection of apoptotic cells

Assessment of apoptotic cells was performed according to published methods (27,28). EA.hy926 cells were exposed to various concentrations of cordycepin (0, 250, 500, 1,000 and 2,000 μg/ml). They were treated with trypsin-EDTA (Sigma-Aldrich) and collected by centrifugation at 150 × g for 10 min, then thoroughly rinsed with PBS. Pellets were resuspended in ice-cold 70% ethanol and fixed at −20°C for 24 h. Cells were then centrifuged (1,000 rpm for 15 min) and ethanol was removed by washing thoroughly with PBS. Cell pellets were resuspended in 1 ml DNA-staining reagent containing 50 μg/ml RNase, 0.1% Triton X-100, 0.1 mmol EDTA (pH 7.4) and 50 μg/ml PI which was provided with the cell apoptosis PI detection kit. Samples were stored in the dark at 4°C for 30 min. Red fluorescence (DNA) was detected through a 563–607 nm band-pass filter using a FC 500 MCL/MPL flow cytometer (Beckman Coulter, Brea, CA, USA). In flow cytometry histograms, apoptotic cells have a signal in the sub-diploid regions, which are well-separated from the normal G1 peak. A total of 10^5^ cells in each sample were analyzed and the percentage of apoptotic cell accumulation in the sub-G1 peak was calculated.

### Transwell migration and invasion assays

A Transwell chamber containing an 8-μm pore polycarbonate membrane filter was coated either with Matrigel (for invasion) or without Matrigel (for migration) and inserted in a 24-well culture plate. HepG2 cells were pre-treated with 0, 125, 250, 500 and 1,000 μg/ml cordycepin for 24 h. The cells were then detached with trypsin-EDTA and resuspended in serum-free DMEM. After filling the lower chamber with media supplemented with 10% FBS as a chemoattractant, 10^5^ cells/well in 0.2 ml serum-free DMEM were loaded in the upper chambers. The apparatus was incubated at 37°C in a humidified chamber with 5% CO_2_ for 12 h (migration assay) or 24 h (invasion assay). Following incubation, the filter was removed. Cells in the upper chamber that did not migrate were scraped away with a cotton swab. The transmembrane cells were fixed in methanol for 30 min, washed twice with PBS and stained with 300 nM DAPI for 5 min. Migrating or invading cells were photographed using an inverted microscope (Axio Observer Z1; Carl Zeiss AG, Oberkochen, Germany) and were counted in five randomly selected fields per membrane, then the averages were calculated. Presented data are representative of three individual wells.

### Wound healing assay

EA.hy926 cells were cultured as confluent monolayers, synchronized in 1% FBS for 24 h and wounded by removing a 300- to 500 μm-wide strip of cells across the well with a standard 200 μl pipette tip. Wounded monolayers were washed twice with PBS to remove non-adherent cells and then treated with 0, 125, 500 and 2,000 μg/ml cordycepin for 6 h. EA.hy926 cell migration was recorded under inverted microscope (Axio Observer Z1; Carl Zeiss AG). Wound healing was quantified, using Image J software (National Institutes of Health, Bethesda, MD, USA), as follows: Wound healing area (%) = [cell-free area (0 h) - cell-free area (6 h)] / cell-free area (0 h) ×100 (29).

### Tube formation assay for angiogenesis

To investigate the effect of cordycepin on angiogenic activity of EA.hy926 cells *in vitro*, a tube formation assay was performed following the procedure by Oikawa *et al* (30). Twenty-four-well cluster tissue culture dishes were coated with 500 μg/ml Matrigel and incubated for 30 min at 37°C. EA.hy926 cells were pre-treated with 0, 125, 250, 500 and 1,000 μg/ml cordycepin for 12 h and were then seeded onto solidified gels at a density of 10^5^ cells/well in 1 ml culture medium. After 24 h of incubation, the total lengths of tube-like structures in five randomly selected microscopic fields per well were determined by phase-contrast microscopy and quantified using Image J software.

### High-performance liquid chromatography (HPLC) assay of intracellular cordycepin levels

Intracellular cordycepin levels were measured according to a previously published method (31). EA.hy926 cells were seeded into six-well plates at a density of 1.5–2×10^6^ cells/well. After reaching confluence, cells were pretreated with 125 μg/ml cordycepin for 0.5–3 h. In order to investigate intracellular cordycepin levels, the culture medium was removed, the cells were rinsed three times with PBS and were submitted to two freeze-and-thaw cycles, then homogenized on ice. The cell homogenate was centrifuged at 12,000 × g for 15 min at 4°C. The supernatant was stored on ice and was filtered through a 0.22-μm filter. The supernatant was finally assayed by HPLC (Dalian Elite Analytical Instruments Co., Ltd., Dalian, China) with dual P230 pumps, an UV230+ detector and analytical software. Samples were processed on an YMC-packed C18 column (5 μm, 250×4.6 mm). The mobile phase consisted of methanol:water (20:80 v/v), with a flow rate of 1.0 ml/min. The UV detector was set at 260 nm and the amount of injected sample was 10 μl. Quantitative analysis of cordycepin was determined by its peak area based on a standard curve built using 100 μg cordycepin. Cordycepin peaks in the samples were identified by the retention time and co-injection tests with the corresponding standard compound. The peak for cordycepin was shown at a retention time of 8.96 min.

### Statistical analysis

All investigations were conducted with at least three independent experiments, each performed in triplicates. Data are expressed as the mean ± standard deviation and were evaluated for statistical significance using one-way analysis of variance followed by Duncan’s multiple range tests. GraphPad Prism 5.0 (GraphPad Software Inc., San Diego, CA, USA) was used to perform statistical analysis. P<0.05 was considered to indicate a statistically significant difference.

## Results

### Cordycepin inhibits EA.hy926 and HepG2 cell proliferation

**Figure 1:**
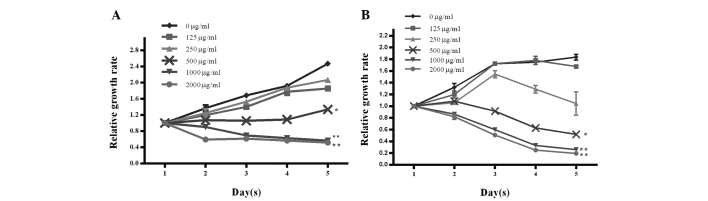
Effects of cordycepin on HepG2 and EA.hy926 cell viability. 3-(4,5-Dimethylthiazol-2-yl)-2,5-diphenyltetrazoliumbromide assay was performed to measure cell viability (the relative growth rate) in (A) EA.hy926 cells and (B) HepG2 cells following treatment with cordycepin at concentrations from 125 to 2,000 μg/ml for 1, 2, 3, 4 and 5 days. EA.hy926 and HepG2 cells treated without cordycepin (0 μg/ml) were used as the negative control. The data are presented as the mean ± standard deviation of three independent experiments. ^*^P<0.05 and ^**^P<0.01, vs. the negative control.

To investigate whether cordycepin affects cell proliferation in HCC cells, we performed MTT assays in EA.hy926 and HepG2 cells. As shown in Fig. 1A and B, the relative growth rates were markedly decreased in the presence of high doses of cordycepin exceeding 500 μg/ml, indicating that cordycepin inhibited EA.hy926 and HepG2 cell proliferation in a dose- and time-dependent manner.

### Cordycepin induces EA.hy926 cell apoptosis

**Figure 2:**
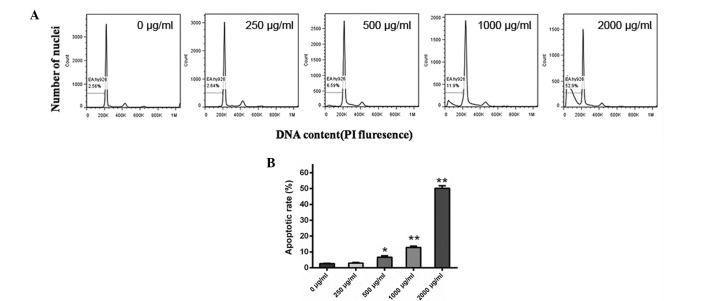
Cordycepin induced apoptosis in EA.hy926 cells. Apoptosis was determined by flow cytometric assay [propidium iodide (PI) staining]. (A) Representative flow cytometric plots. EA.hy926 cells were treated with various concentrations of cordycepin (0, 250, 500, 1,000 and 2,000 μg/ml) for 24 h. (B) The number of apoptotic cells divided by the total number of cells (counted manually), expressed as the percentage of total cells. The data are presented as the mean ± standard deviation of three independent experiments. ^*^P<0.05 and ^**^P<0.01, vs. the negative control (0 μg/ml).

To assess whether cordycepin affects apoptosis of endothelial cells, we incubated EA.hy926 cells with 0, 250, 500, 1,000 and 2,000 μg/ml cordycepin for 24 h and performed a flow cytometry assay. As shown in Fig. 2A and B, 250 μg/ml cordycepin had no detectable effect on cell apoptosis, while 500, 1,000 and particularly 2,000 μg/ml cordycepin caused a marked increase in the percentage of apoptotic cells, compared with the negative control (0 μg/ml) (all P<0.05).

### Corcydepin inhibits HepG2 cell migration and invasion

**Figure 3:**
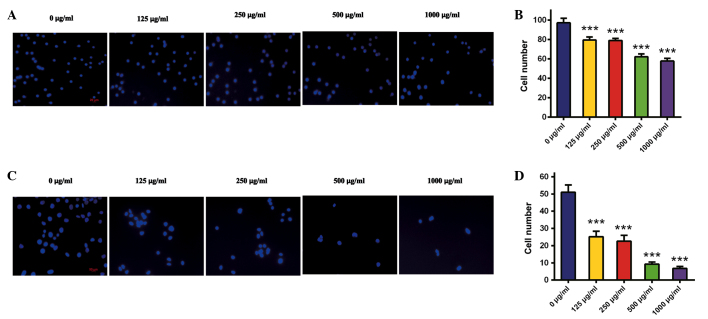
Effects of cordycepin on migration and invasion of HepG2 cells. The migrating and invading abilities of HepG2 cells were examined by Transwell chamber assay. HepG2 cells were treated with various concentrations of cordycepin (0, 125, 250, 500 and 1,000 μg/ml) for 12 h (migration assay) or 24 h (invasion assay). (A and C) Migrating or invading cells were photographed under an inverted fluorescence microscope (A, ×100; C, ×200). Blue represents DAPI staining. (B and D) Quantification of the numbers of migrating or invading cells are presented as the mean ± standard deviation of three independent experiments performed in triplicate. ^*^P<0.05 and ^**^P<0.01, vs. the negative control (0 μg/ml).

In HCC development and metastatic spread, cell migration and invasion are essential processes. To detect antitumor activities of cordycepin on HepG2 cells, Transwell migration and invasion assays either without or with precoated Matrigel were performed following treatment with cordycepin at doses of 0, 125, 250, 500 and 1,000 μg/ml. HepG2 migration was significantly suppressed by cordycepin in a dose-dependent manner (all P<0.05; Fig. 3A and B). The numbers of migrating cells following treatment with cordycepin at doses of 0, 125, 250, 500 and 1,000 μg/ml were 97±5, 80±3, 79±2, 62±3 and 58±3, respectively.

Similarly, HepG2 cell invasion was markedly inhibited in a dose-dependent manner (all P<0.05; Fig. 3C and D). The numbers of invading cells following incubation with 0, 125, 250, 500 and 1,000 μg/ml cordycepin were 51±4, 25±3, 23±3, 9±1 and 7±1, respectively. These results demonstrate that cordycepin significantly reduced the potential of HepG2 cells to migrate and invade.

### Cordycepin inhibits EA.hy926 cell migration and angiogenesis

**Figure 4:**
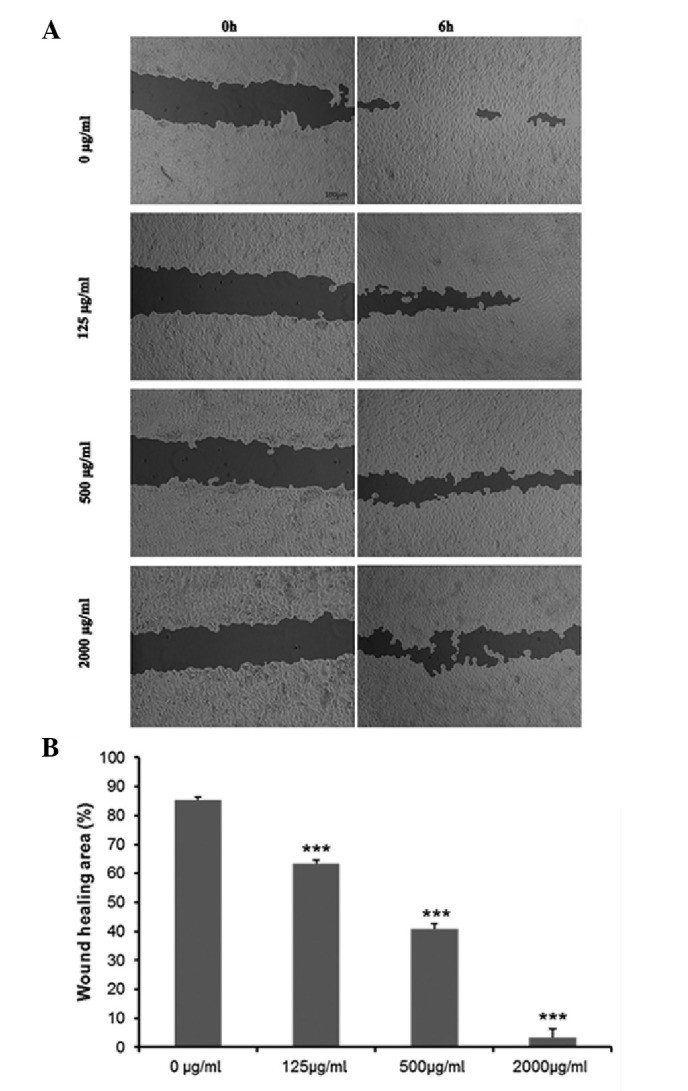
Effect of cordycepin on the migration of EA.hy926 cells by wound healing assay. EA.hy926 cells were treated with various concentrations of cordycepin (0, 125, 500 and 2,000 μg/ml) for 6 h. (A) Migration of EA.hy926 cells were photographed under a light microscope (scale bar, 100 μm) and the data were digitally recorded. (B) The percentages of wound healing area are presented as the means ± standard deviation of three independent experiments. ^***^P<0.001, vs. the negative control (0 μg/ml).

To explore whether cordycepin affects the angiogenic potential of HCC cells, we examined cell migration and angiogenesis by wound healing and tube formation assays in EA.hy926 cells. The wound healing assay demonstrated that the migration of EA.hy926 cells incubated with cordycepin was significantly decreased compared with the negative control (0 μg/ml) (all P<0.05). After treatment with various concentrations of cordycepin for 24 h, the percentage of wound healing area was 85.48±0.84% in the negative control cells, 63.50±1.08% in 125 μg/ml-treated cells, 40.81±1.76 in 500 μg/ml-treated cells and 3.45±0.29% in 2,000 μg/ml-treated cells (Fig. 4A and B).

**Figure 5:**
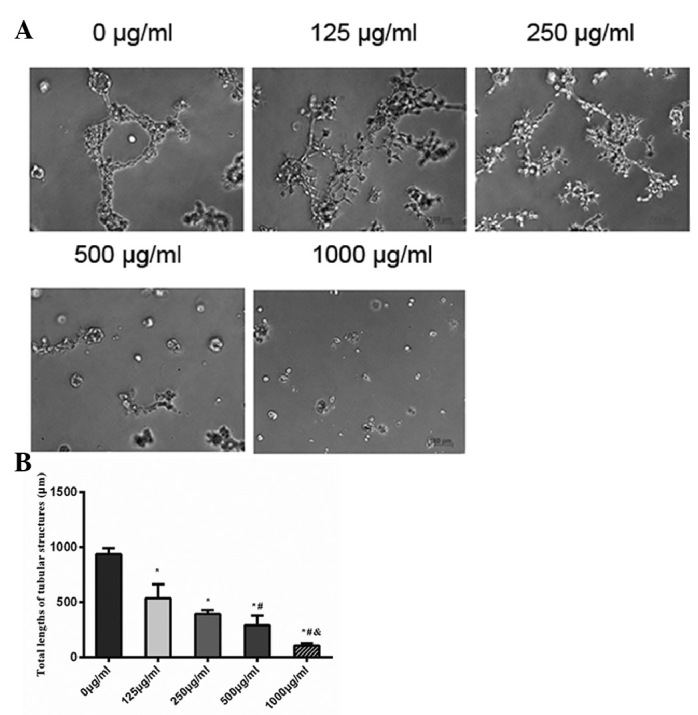
Cordycepin inhibited tube formation of EA.hy926 cells. EA.hy926 cells were treated with different concentrations of cordycepin (0, 125, 250, 500 and 1,000 g/ml) and added to 24-well plates precoated with Matrigel for 24 h. (A) Tube formation of EA.hy926 cells were photographed under a light microscope (magnification, ×100). (B) Quantification of the total length of tubular structure under different cordycepin concentrations. ^*^P<0.05, vs. the negative control (0 μg/ml); ^#^P<0.05, vs. cells treated with 125 μg/ml cordycepin; ^&^P<0.05, vs. cells treated with 250 μg/ml cordycepin.

As shown in Fig. 5A, EA.hy926 cells in the negative control aligned to form tube-like structures and crossing tubes with multicentric junctions. EA.hy926 cells treated with various concentrations of cordycepin tended to form fewer tubes, as well as fewer and weaker junctions. The total lengths of tubular structure after incubation with 0, 125, 250, 500 and 1,000 μg/ml cordycepin for 24 h were 936±56, 536±126, 395±31, 292±88 and 107±39 μm (Fig. 5B). The results revealed that cordycepin significantly inhibited migration and angiogenesis of endothelial cells.

### Intracellular cordycepin level was stable in EA.hy926 cells

**Figure 6:**
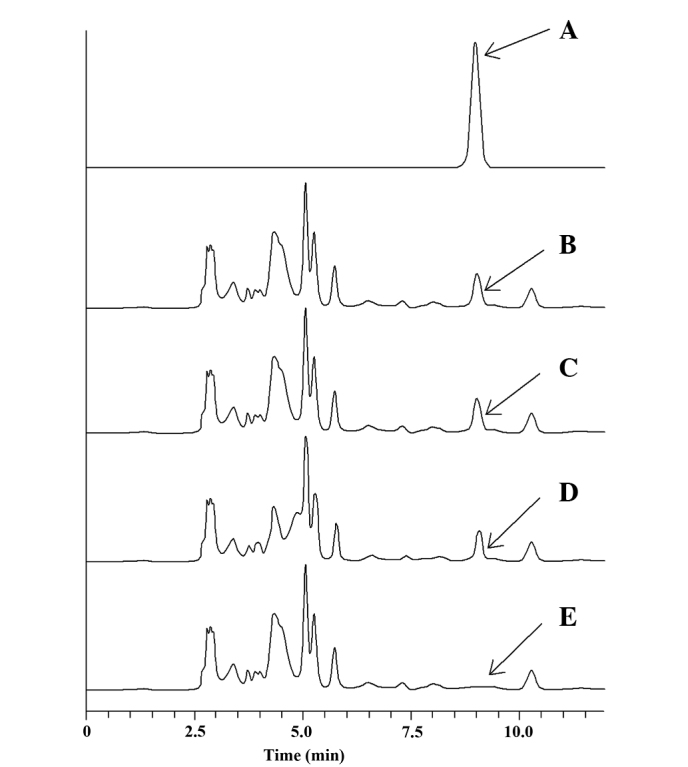
Intracellular cordycepin concentrations in EA.hy926 endothelial cells quantified by high-performance liquid chromatography. Under the chromatographic conditions used, cordycepin had a retention time of 8.96 min. (A) Cordycepin standard curve (100 μg/ml). (B) Intracellular concentration of cordycepin after 0.5 h. (C) Intracellular concentration of cordycepin after 1 h. (D) Intracellular concentration of cordycepin after 3 h. (E) Cytosol of EA.hy926 cells without cordycepin. Arrows show the locations of cordycepin in chromatographic graphs.

Based on the standard curve of cordycepin, the stability of intracellular cordycepin is shown in Fig. 6A. EA.hy926 cells were incubated with the culture medium (DMEM plus 10% FBS) containing 125 μg/ml cordycepin from 0.5 to 3 h (Fig. 6B–D). The cytosol of EA.hy926 cells without cordycepin is shown in Fig. 6E. The cellular content was assayed by HPLC. Under the chromatographic conditions used, cordycepin had a retention time of 8.96 min. The results demonstrated that cordycepin was able to permeate the cell membrane of EA.hy926 cells and was stable during the 3 h of incubation.

## Discussion

The present study demonstrated that cordycepin extracted from *C. militaris* inhibited HepG2 cell proliferation, migration and invasion. Simultaneously, cordycepin also inhibited vascular endothelial EA.hy926 cell proliferation, migration and angiogenesis, and induced apoptosis. Therefore, cordycepin targeting tumor and endothelial cells may promote the efficacy of therapy in HCC.

*C. militaris*, from which cordycepin is extracted, has long been used in traditional Chinese medicine (9). Cordycepin exerts numerous pharmacological actions, such as suppression of cell proliferation, activation of apoptosis, and inhibition of cell migration and invasiveness in different tumor cell lines (15,32–35). Cordycepin reduced metastatic nodule formation in mice (34) and has therefore been proposed as an antimetastatic agent. The effects of cordycepin are mainly due to the inhibition of polyadenylation and the activation of AMP-activated protein kinase in the mTOR signaling pathway, in doses over 200 μM (24,36). However, only a few reports have focused on the effects of cordycepin on cell proliferation, migration and invasion in HCC cells. The ability of HCC cells to endlessly proliferate is mainly associated with the deregulation of the cell cycle and promotion of invasion. Previous studies suggested that cordycepin reduces lipid deposition and cholesterol levels in HepG2 cells, but has no effect on cell proliferation, and suggested that cordycepin may have a protective effect on the liver (37,38). In an additional study, pure cordycepin at concentrations of 100 μM had no inhibitory effects on HepG2 cells and no potent *in vitro* cytotoxicity (39). However, studies performed in other HCC cell lines, such as BEL-7402 (21), Hep3B (22) and rat H4 (23) showed results similar to those observed in the present study. Our results also indicated that cordycepin exerts an anti-invasive cytotoxic action in HepG2 cells, and that this effect may contribute, at least in part, to the antimetastatic effect observed in previous studies.

A number of studies have indicated that blood vessel proliferation in a tumor is a hallmark of tumor growth and metastatic spread (40,41). HCC tumor vasculature shows irregular diameter and an abnormal vascular branching pattern; these tumor vessels also typically lack a complete basal membrane and are incompletely covered by pericytes and are therefore leaky (7). Cancer cells can spontaneously fuse with endothelial cells to form hybrid cells, facilitating the invasion of the endothelial barrier to form metastases (42). Since HCC is a hypervascular tumor, uncontrolled angiogenesis plays an important role in HCC development, and thereby anti-angiogenic agents became one of the most promising therapeutic strategies in HCC (43). In our study, we explored the effect of cordycepin on angiogenesis of immortalized human umbilical vein endothelial cells (EA.hy926). These cells are the product of the fusion between human umbilical vein cells and a thioguanine-resistant A549 clone. These cells show morphological, phenotypic and functional characteristics of human endothelial cells, without the limited lifespan and the inter-donors variability. These cells are considered good models for cancer drug screening (44,45). As expected, the results of the present study demonstrated that cordycepin effectively inhibits vascular endothelial cell growth and induces apoptosis. Moreover, it was observed that the anticancer effects of cordycepin are likely to be associated with inhibition of endothelial cell migration and tube formation. Therefore, our results suggested that cordycepin has potential antiangiogenic activity. The effects observed at low doses may be due to decreased polyadenylation of mRNAs, while the effects observed at high doses may be due to the activation of the mTOR pathway (24,36).

A previous study demonstrated that cordycepin exerts its effects at doses over 200 μM (24). Additionally, pharmacokinetic data demonstrated that cordycepin has a short half-life and is metabolized in a short period of time. As an adenosine analog, the metabolic pathway for cordycepin may be similar to adenosine. Cordycepin is rapidly deaminated by adenosine deaminase, and is promptly metabolized to an inactive metabolite, 3′-deoxy-hypoxanthinosine (46,47). The half-life of cordycepin in rat blood is 1.6±0.0 min after administration, and the measurable concentration of cordycepin in rat blood vanishes within 30 min. Cordycepin-induced compounds appear in the blood and liver for over 2 h after administration (11). To overcome the problem of rapid elimination, a high dosage must be administered; otherwise, intracellular concentrations would be sub-therapeutic. Hence, the present study used cordycepin at doses ranging from 125 to 2,000 μg/ml, which allowed detection and therapeutic effects. Using a HPLC method, we showed that cordycepin is able to permeate the EA.hy926 cell membrane within 0.5 h and was stable for the whole studied period (3 h). Thus, local administration of high doses of cordycepin may be sufficiently potent and specific to destroy enough tumor vasculature to starve the entire tumor.

In conclusion, our results indicate that cordycepin possesses anticancer properties, which are not solely a result of direct cytotoxicity in a HCC cellular model, but also of inhibition of angiogenesis in vascular endothelial cells. Our results also suggest that a dose greater than 500 μg/ml is required in order to observe therapeutic effects. Cordycepin may be a potential anti-angiogenic candidate for cancer therapy in HCC; however, its mechanisms and adverse effects require further investigation.
